# High level of plasma TILRR protein is associated with faster HIV seroconversion

**DOI:** 10.1016/j.ebiom.2022.103955

**Published:** 2022-03-24

**Authors:** Mohammad Abul Kashem, Jennifer Lischynski, Brittany Stojak, Lin Li, Xin-Yong Yuan, Binhua Liang, Joshua Kimani, Francis A Plummer, Ma Luo

**Affiliations:** a Department of Medical Microbiology and Infectious Diseases, University of Manitoba, Winnipeg, MB, Canada; b JC Wilt Infectious Diseases Research Centre, National Microbiology Laboratory, Public Health Agency of Canada, Winnipeg, MB, Canada; c Department of Microbiology and Veterinary Public Health, Chittagong Veterinary and Animal Sciences University, Chittagong, Bangladesh; d National Microbiology Laboratory, Public Health Agency of Canada, Winnipeg, MB, Canada; e The Max Rady College of Medicine, University of Manitoba, Winnipeg, MB, Canada; f Department of Biochemistry and Medical Genetics, University of Manitoba, Winnipeg, MB, Canada; g Institute for Tropical and Infectious Diseases, University of Nairobi, Nairobi, Kenya; h Center for STD/HIV Research & Training, University of Nairobi

**Keywords:** TILRR, Blood plasma, Proinflammatory mediators, HIV seroconversion, Women, Pumwani sex worker cohort

## Abstract

**Background:**

TILRR (Toll-like Interleukin-1 Receptor Regulator) is a modulator of many genes in NF-κB (nuclear factor kappa-light-chain-enhancer of activated B cells) signaling. It promotes the production of inflammatory mediators and the migration of immune cells. Recently, we showed that TILRR protein circulates in human blood. Thus, it could influence systemic inflammation. Systemic and mucosal inflammations increase the susceptibility to HIV infection. In this study, we analyzed the TILRR protein levels of the archived plasma samples of women enrolled in the Pumwani cohort to determine whether the plasma TILRR protein levels before seroconversion are correlated with differential risk of HIV seroconversion.

**Methods:**

TILRR protein of 941 archived HIV negative plasma samples from 390 women who were HIV negative at the cohort enrollment was quantified with an in-house developed multiplex bead array method. Proinflammatory cytokines/chemokines were measured using a 14-plex bead array method. Spearman rank correlation analysis was used to determine the correlation between plasma TILRR protein and proinflammatory cytokines/chemokines. Kaplan-Meier survival analysis was conducted to evaluate whether the median plasma TILRR protein levels correlate with increased risk of HIV seroconversion.

**Findings:**

The level of plasma TILRR protein was positively correlated with plasma IL-1β (rho: 0.2593, *p*<0.0001), MCP-1 (rho: 0.2377, *p*<0.0001), and IL-17A (rho: 0.1225, *p*=0.0216). Women with median plasma TILRR protein levels ≥100 ng/ml seroconverted significantly faster than women with plasma TILRR protein levels <100 ng/ml (log-rank= 100.124, *p*<0.0001; relative risk= 3.72 and odds ratio= 15.29). Furthermore, the factors causing genital inflammation, such as STIs (sexually transmitted infections), vaginal discharge, and genital ulcers were not statistically significantly different among women with different median plasma TILRR protein levels.

**Interpretation:**

The high plasma TILRR protein levels are highly correlated with several plasma proinflammatory cytokines/chemokines. High median plasma TILRR protein (≥100 ng/ml) strongly predicted an increased risk of HIV seroconversion. Reducing plasma TILRR protein levels may reduce the risk of HIV acquisition.

**Funding:**

The study was funded by an operating grant from the Canadian Institutes of Health Research (CIHR), operating grant-PA: CHVI Vaccine Discovery and Social Research (http://www.cihr-irsc.gc.ca/e/193.html), and National Microbiology Laboratory of Canada.

**Research in context**

**Evidence before this study**

Previous studies showed that TILRR increases the expression of many genes in the NF-κB signal transduction pathway and regulates inflammatory responses. It increases the production of inflammatory mediators and promotes the migration of immune cells, including HIV target cells, towards the TILRR-overexpressed HeLa cell culture supernatants. TILRR is also found to be involved in the development of atherosclerosis and breast cancer. We recently found that TILRR protein circulates in the blood.

**Added value of this study**

This study showed that plasma TILRR protein levels positively correlated with several plasma proinflammatory mediators, and high plasma TILRR protein levels strongly predicted an increased risk of HIV seroconversion in the Pumwani sex worker cohort. Thus, the study represents an important advance in identifying risk factors of HIV acquisition.

**Implications of all the available evidence**

Plasma TILRR protein is an important modulator of systemic inflammation, and plasma TILRR protein level is a strong predictor of an increased risk of HIV acquisition. Plasma TILRR protein represents an important target to reduce systemic inflammation and the risk of HIV acquisition.

Alt-text: Unlabelled box

## Introduction

TILRR (Toll-like Interleukin-1 Receptor Regulator) is a splice variant of FREM1 (FRAS-related Extracellular Matrix 1).^1^ It has been identified as a co-receptor for IL-1R1 (interleukin-1 receptor type 1) and potentiates MYD88 (myeloid differentiation primary response 88) recruitment to control Ras-dependent amplification of NF-κB (nuclear factor kappa-light-chain-enhancer of activated B cells).^1^ Our studies showed that TILRR is a major modulator of many inflammatory responsive genes in the NF-κB signal transduction pathway, increases chemokine and cytokine secretion by epithelial cells, and promotes migration of immune cells.^2^^,^^3^ Inflammation promotes activation and localization of HIV target cells, and elevated cervicovaginal inflammation has been associated with a higher risk of HIV acquisition.^4^, ^5^, ^6^, ^7^, ^8^, ^9^

Although it is well known that systemic and mucosal inflammation are risk factors for HIV acquisition and disease progression, the potential involvement of TILRR with HIV infection comes from studies of a group of HIV resistant female sex workers in the Pumwani sex worker cohort (PSWC).^10^ The Pumwani cohort was established in Nairobi, Kenya in 1985 to study sexually transmitted infections.^11^^,^^12^ Long-term bi-annual clinical follow-ups identified a sub-group of sex workers who remain HIV uninfected despite active high-risk sex work.^13^ Studies have shown that resistance to HIV infection observed in these women does not correlate with altered sex practices or behavioral differences. This natural resistance to HIV is multi-factorial and the result of a combination of host genetics and innate and adaptive immune responses.^14^, ^15^, ^16^, ^17^, ^18^ A low-resolution genome-wide SNP (single nucleotide polymorphism) analysis showed that the minor allele of a SNP rs1552896 in the FREM1 gene was significantly enriched in HIV-resistant women, and the major allele is associated with increased susceptibility to HIV acquisition.^10^ Our subsequent studies showed that the FREM1 splice variant mRNA encoding TILRR protein was rarely detected in PBMCs of women who have the minor allele of SNP rs1552896 but was abundant in women who were homozygous for the major allele.^19^

Recently, we found that TILRR protein is not only expressed in cells and tissues but also circulates in the blood.^20^ Because TILRR protein regulates NF-κB inflammatory responses,^2^ promotes the production of inflammatory mediators,^2^ and immune cell migration,^3^ the plasma TILRR protein may regulate systemic inflammation. In this study, we evaluated plasma TILRR protein level, its correlation with plasma proinflammatory cytokines/chemokines, and the risk of HIV acquisition in the Pumwani sex worker cohort. We showed that high median plasma TILRR protein level positively correlated with IL-1β (interleukin-1 beta), MCP-1 (monocyte chemoattractant protein-1), and IL-17A and strongly predicted the risk of HIV seroconversion in the Pumwani cohort.

## Methods

### Study subjects

The Pumwani sex worker cohort is an open prospective cohort established in 1985 to study the epidemiology and immunobiology of STIs (sexually transmitted infections) including HIV. Women, who came to the research clinic for STIs and HIV prevention care, including the provision of free condoms, consultation, and treatment of other infections, were voluntarily enrolled into the cohort after informed written consent.^13^^,^^21^^,^^22^ All women enrolled in the cohort were screened for anti-HIV p24 using enzyme immunoassay (EIA). HIV seroconversion was confirmed by either Novapath Immunoblot HIV-1 (Bio-Rad) or Recombigen HIV-1/2 EIA (Cambridge, Biotech). Women who were negative for antibodies on EIA were further confirmed by immunoblot testing. All participants were routinely screened for HIV infection by PCR (polymerase chain reaction) assay. The women enrolled in the cohort have been followed biannually since the cohort establishment in 1985.^11^ Many women who were HIV negative at enrollment were seroconverted within the first three years between 1985 and 1995.^13^ The time of HIV seroconversion was estimated based on the mid-point between the last seronegative test and the first seropositive test. A subgroup of women remained HIV negative despite active sex work. This group of women was defined as HIV resistant and they all enrolled in the cohort before 2000 and have a follow-up time of 9.56±4.37 years before 2006. All women who were HIV uninfected at the cohort enrolment between 1985 and 2008 and have available plasma samples collected before seroconversion in the sample biorepository were included in this study. The 390 women and 941 HIV negative plasma samples included in this study represent all the available archived HIV negative plasma samples of women who were HIV negative at the cohort enrollment.

### Ethics statement

This study was guided by the Helsinki Declaration on ethical principles for medical research involving human subjects. Ethics approval to conduct this study using human plasma samples was obtained from the University of Manitoba and the University of Nairobi/Kenyatta National Hospital Ethics Committee (KNH/ERC/R/169). All enrolled subjects gave written informed consent to participate in the study.

### Sample-size and power calculation

This study analyzed 941 available archived HIV-negative plasma samples from 390 subjects who were HIV negative at the cohort enrollment. Whenever available, plasma samples from multiple visits were included in the analysis to evaluate the fluctuations of plasma TILRR protein of subjects over time. Based on all available plasma samples that fit the study criteria, such as HIV negative plasma samples from women who were HIV negative at cohort enrollment (*n*= 390), we conducted a power calculation between two independent study groups using an online post-hoc power calculator (https://clincalc.com/stats/power.aspx). The study groups were binned based on the plasma TILRR levels (group 1: 0-99 ng/ml [*n*= 285; HIV infection risk: 21.75%] and group 2: ≥100 ng/ml [*n*= 105, HIV infection risk: 80.95%]). The calculation was performed to estimate the 80–100% study power using a type I error rate (α-value) of <0.0001. The power analysis with the population sample size of 390 showed a >99% statistical study power (Table S1).

### Preparation of plasma samples

A total of 941 HIV-negative plasma samples of 390 women enrolled in the Pumwani cohort between 1985 and 2008 were included in this study. Samples were prepared as described previously.^20^ Briefly, the samples were pulled from -80^0^ C freezers and kept on ice to thaw. Following centrifugation at 10,000xg for 10 min at 4^0^ C, the supernatants were collected and diluted (1:10) with sample diluents (PBS pH 7.2 containing 2 mM EDTA, 150 mM NaCl, 1% IGEPAL CA-630, and 20 mM Tris pH 8.0). In total, 120 µl samples (12 µl plasma + 108 µl of sample diluents) were prepared to use as duplicates for each participant's plasma samples. All diluted samples were kept on ice and vortexed for 15 sec before being added to the assay plate.

### Measurement of TILRR protein in plasma samples

TILRR protein in plasma samples was measured by the in-house developed protocol as described previously.^20^ In brief, the assay was conducted using a 3-plex anti-FREM1 mAbs panel. The anti-FREM1 mAbs coupled magnetic bead stock (5 × 10^6^/ml) was diluted at 1:100 in custom buffer (PBS pH 7.2 containing 2 mM EDTA, 150 mM NaCl, and 1% IGEPAL CA-630), and dispensed 50 µl of beads to each well of the 96-well assay plate. After 2X wash with wash buffer, 50 µl plasma samples (1:10 dilutions) were added to the wells in duplicates and incubated for 2h at room temperature (RT) on a plate shaker (850±50 rpm). After 3X washes, 30 µl of the detection antibody (1 µg/ml) were added and incubated again for 30 min at RT on a plate shaker. After 3X washes, 50 µl streptavidin-PE conjugate (1X) was added and incubated for 10 min at RT on a shaker. Finally, 125 µl PBS (pH 7.2) was added to each well, and data was acquired by Bio-Plex^TM^ 200 System (Bio-Rad). To generate standard curve, 50 µl of rFREM1 spD protein (1:5 dilutions in PBS pH 7.2 containing 2 mM EDTA, 150 mM NaCl, and 1% IGEPAL CA-630) was added in 7 wells in duplicates. For background control, 50 µl of dilution buffer (standard diluent) was added in duplicates. Bio-Plex software version 6.1 was used to acquire the data. The data were optimized to calculate the Upper limit of quantification (ULOQ, ng/ml) and Lower limit of quantification (LLOQ, ng/ml) using logistic-5PL regression analysis with fitness probability maintained ≥ 0.95. All anti-TILRR antibodies and reagents used in this study were validated by our earlier studies.^20^^,^^23^

### Measurement of cytokines/chemokines in the corresponding plasma samples

We analyzed the cytokines/chemokines in plasma samples using an in-house developed 14-plex Bio-Plex assay as described earlier.^2^^,^^24^ The cytokines and chemokines include granulocyte-macrophage colony-stimulating factor (GM-CSF), interferon-gamma (IFNγ), interleukin (IL)-1α, IL-1β, IL-6, IL-8/CXCL8, IL-10, IL-17A, IFN-γ inducible protein (IP)-10/CXCL10, monocyte chemoattractant protein (MCP)-1/CCL-2, monocyte inflammatory protein (MIP)-1α, MIP-1β, regulated upon activation, normal T cell expressed and secreted (RANTES)/CCL5, and tumor necrosis factor-alpha (TNF-α). Briefly, antibody-coupled bead stocks (14-plex) were combined at 1:600 dilutions in assay buffer (phosphate-buffered saline+1% bovine serum albumin). Plasma samples were diluted to 1:27 in assay buffer. Twenty-five microliter of combined detection antibodies (1 µg/ml each) was added to each well. A custom 14-plex human cytokine/chemokine protein standard was used in the assay (Table S2). The overall assay and data analysis were conducted based on the protocol as described for the measurement of plasma TILRR protein. Antibodies and reagents used in the 14-plex assay were validated by our previous studies.^25^^,^^26^

### Statistical analysis

SPSS (Statistical Package for Social Science, IBM, USA) was used for data analysis, including Spearman rank correlation analysis, generating median plasma TILRR protein level for participants with data of multiple samples from multiple visits, and the Kaplan-Meier analysis (log rank [Mantel-Cox] test, relative risk [RR], odds ratio [OR], and Cox regression hazard ratio [HR] with 95% confidence interval [CI]) of the effect of median plasma TILRR protein level on HIV seroconversion. Spearman rank analysis was performed to analyze the correlation between the level of plasma TILRR protein and the level of plasma inflammatory mediators.

Kaplan-Meier survival analysis was conducted to examine the influence of the plasma TILRR protein levels on HIV seroconversion. For Kaplan-Meier analysis, median plasma TILRR protein data were first generated from each participant using SPSS. We then plotted the distribution of the median plasma TILRR level of all participants. Based on the distribution of the median plasma TILRR protein level, we arbitrarily divided the participants into 4-groups (0-99 ng/ml, 100-499 ng/ml, 500-999 ng/ml, and ≥1000 ng/ml). Kaplan-Meier analysis was conducted to determine the time to seroconversion for the 4 groups of participants with different levels of plasma TILRR protein. The analysis showed that three participant groups with plasma TILRR levels ≥100 ng/ml were seroconverted much faster than women who had the lowest level of plasma TILRR protein (<100 ng/ml). We then combined all participants with plasma TILRR levels ≥100 ng/ml into one group and conducted further Kaplan-Meier survival analysis (0-99 ng/ml and ≥100 ng/ml) using log rank test. RR and OR were calculated from the case summary of Kaplan-Meier analysis using an online calculator (https://www.socscistatistics.com/biostatistics/default2.aspx).

One-way ANOVA with Bonferroni multiple comparison tests were used to analyze the age and duration of sex work of the participants in the different plasma TILRR groups (0-99 ng/ml, 100-499 ng/ml, 500-999 ng/ml, and ≥1000 ng/ml). Since the data for age and duration of sex were not equal, we applied one-way ANOVA for multiple comparisons. The Chi-square test was performed for the analysis of potential confounding factors (categorical), including STIs, vaginal discharge, and genital ulcers. All *p*<0.05 were reported as statistically significant. There was no multiplicity issue in this study.

### Role of funders

The funders of this study had no role in study design, collection of samples, data analysis, and data interpretation, in the writing of the report or decision to submit for publication. The corresponding author had full access to all data in this study and had final responsibility for the decision to submit for publication.

## Results

### Demographic characteristics of the study subjects

In this study, we examined 941 HIV-negative archived plasma samples from 390 women who were HIV-negative at the cohort enrollment. The detailed demographic characteristics of all study participants were shown in Table 1. The median age (interquartile range, IQR) of the study subjects at the time of sample collection was 33.00 (28.00–38.50) years old. The median (IQR) duration of sex work for the study subjects was recorded as 8.00 (4.00–14.00) years. The women enrolled in the Pumwani sex worker cohort were from small towns of rural Kenya (64.12%) Tanzania (32.72%), and Uganda (3.17%). Approximately 90% of them were Bantu speakers and 10% of study subjects were Nilos speakers. Sexually transmitted infections (STIs) were found at 44.65% (171/383). STIs were considered positive when participants were diagnosed with any one of the following disease conditions: gonorrhea, syphilis, chlamydial infection, and bacterial vaginosis. In addition, some subjects were reported to have vaginal discharge and genital ulcer at the time of sample collection, which was estimated at 30.81% (118/383), and 11.75% (45/383), respectively.

**Table 1:** Demographic characteristics of the study subjects.

Characteristics		Study subjectsMedian (IQR)
Age at sample collection, in years		33.00 (28.00-38.50) (n=370^a^)
Duration of sex work at sample collection, in years		8.00 (4.00-14.00) (n=358^b^)

		**% (n)**
Nationality	Kenya	64.12 (243/379^c^)
	Tanzania	32.72 (124/379^c^)
	Uganda	3.17 (12/379^c^)
Language	Bantu speakers	90.24 (342/379^c^)
	Nilos speakers	9.76 (37/379^c^)
Sexually transmitted infection (STIs)		44.65 (171/383^d^)
Vaginal discharge		30.81 (118/383^d^)
Genital ulcer		11.75 (45/383^d^)

a Women with known age at the time of collection. Age was unknown for 20-women.

b Women with known duration of sex work at the time of collection. Duration of sex work was unknown for 32-women.

c Women with known nationality and language. Nationality and language were unknown for 11-women.

d Samples with a history of known STIs (Gonorrhea, Syphilis, Chlamydial infection, and bacterial vaginosis), vaginal discharge, and genital ulcer. Six women did not have a history of STIs, vaginal discharge, and genital ulcers. IQR, interquartile range; n, subject#.

### Frequency of TILRR protein detection in the archived plasma samples

**Figure 1:**
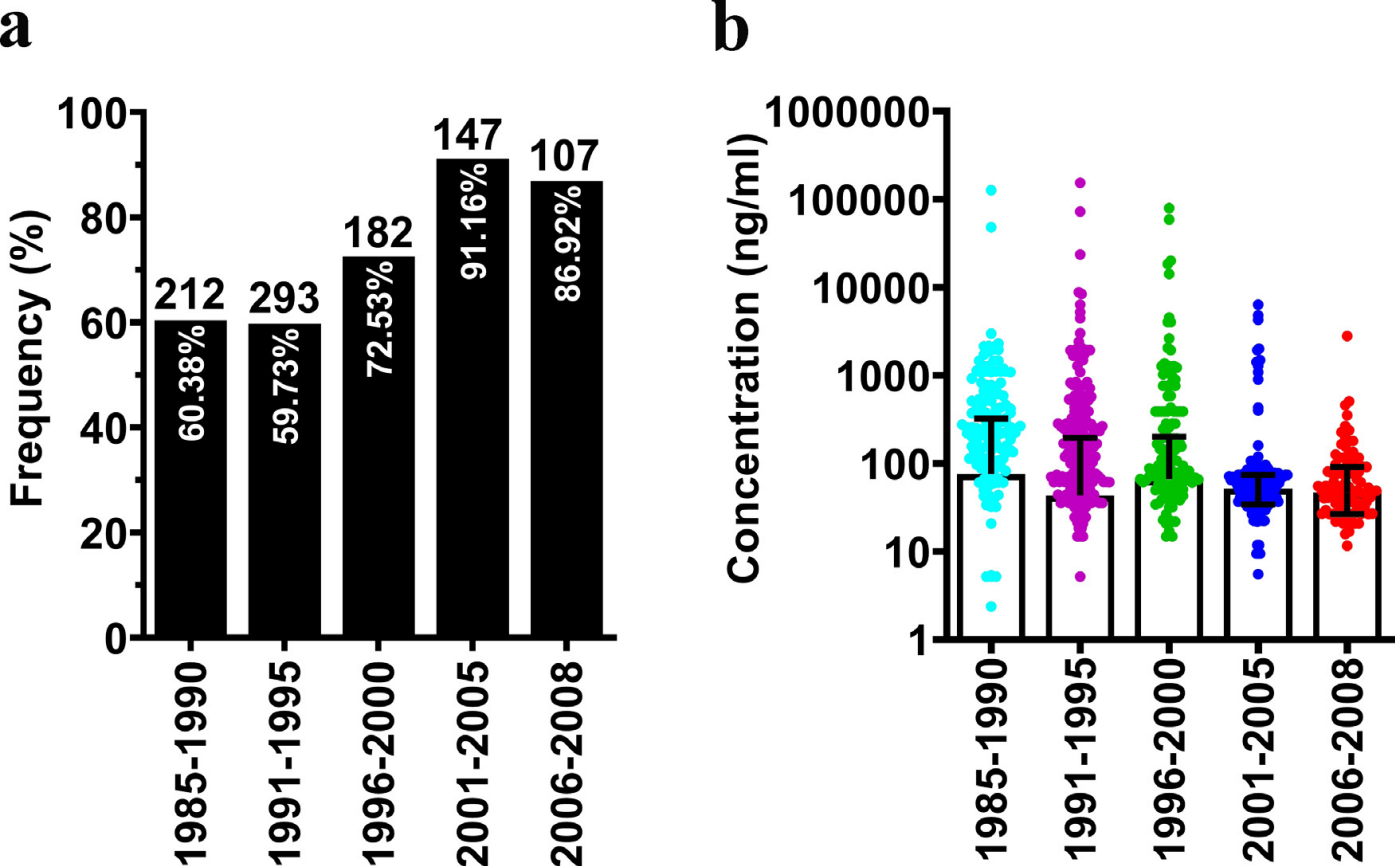
Overall frequency and the level of TILRR protein in archived plasma samples of all participants of PSWC. (a) Frequency of plasma TILRR protein detection in blood plasma among participants collected between 1985 and 2008 (*n*= 941); (b) The level of TILRR protein (median with IQR) in plasma samples collected between 1985 and 2008 (*n*= 941). The detailed data are presented in Figures S2, 3, Tables S3–5. The number above and within each bar of Figure a represents the total samples (n) tested and the plasma TILRR protein detection frequency (%), respectively.

To assess whether the archived plasma samples (n= 941) are suitable for the analysis of TILRR protein, we analyzed the overall detection frequency of TILRR protein in plasma samples collected between 1985 and 2008. The analysis showed that overall detection frequency was higher in plasma samples collected between 2001 and 2008 (Figure 1a and Table S3; a detailed breakdown is presented in Figure S1a, b and Table S4). More specifically, the highest detection frequency was recorded as 91.16% (134/147) between 2001 and 2005, and the lowest detection frequency was 59.73% (175/293) from 1991 to 1995. The frequency of 60.38% (128/212), 72.53% (132/182), and 86.92% (93/107) was observed during 1985-1990, 1996-2000, and 2006-2008, respectively. To exclude the possibility that sample storage conditions may influence TILRR detection frequencies and levels, we analyzed samples collected from the same individuals over years. The data showed that there are very little variations during the years for samples collected over years of several individuals (Figure S2a–c and Table S5). These data showed that the archived plasma samples are suitable for further analysis.

Next, we analyzed whether the level of TILRR protein in plasma was correlated with the frequency of detection in the archived plasma samples. The analysis showed that the highest level of TILRR protein was observed in samples collected between 1985 and 1990 (median [IQR]: 75.65 [0-317.02] ng/ml) whereas the lowest level of TILRR protein was observed in plasma samples collected during 1991-1995 (median [IQR]: 43.36 [0-194.63] ng/ml) (Figure 1b and Table S3). Thus, the levels of TILRR protein in the archived plasma samples are not correlated with the frequencies of TILRR protein detection and the plasma samples collected between 1985 and 2008 are suitable for further analysis with other factors, such as plasma inflammatory cytokines/chemokines and HIV seroconversion.

### Plasma TILRR protein level is positively correlated with the levels of several plasma inflammatory cytokines/chemokines

Next, we examined the plasma TILRR protein level and its relationship with the levels of 14 plasma inflammatory cytokines/chemokines in 352 samples. Spearman rank correlation analysis was conducted and showed that plasma TILRR level is positively correlated with the levels of several plasma proinflammatory cytokines/chemokines, including IL-1β (rho= 0.2593, *p*<0.0001), MCP-1 (rho= 0.2377, *p*<0.0001), and IL-17A (rho= 0.1225, *p*=0.0216) (Tables 2 and S6). There is also a trend of positive correlation of plasma TILRR with the levels of IFNγ, IL-6, GM-CSF, MIP-1α, MIP-1β, IL-10, and IP-10. Many of the proinflammatory cytokines/chemokines were positively correlated with each other (Tables 2 and S6). For instance, IL-1β was positively correlated with IL-6, GM-CSF, MCP-1, MIP-α, MIP-β, IL-10, IP-10, and IL-17A. MCP-1 was also significantly correlated with IL-6, GM-CSF, IL-1β, MIP-α, MIP-1β, IP-10, and IL-17A. Furthermore, IFNγ, IL-6, GM-CSF, MIP-1α, MIP-1β, IL-10, IP-10, and IL-17A were also positively correlated with each other.

**Table 2:** Spearman rank correlation of plasma TILRR protein level with plasma inflammatory cytokines/chemokines.

	TILRR	IFNγ	IL-6	GM-CSF	IL-1β	MCP-1	MIP-1α	MIP-1β	IL-10	IP-10	IL-17A
**TILRR**	1.0000	0.0972	0.0990	0.0152	0.2593	0.2377	0.0166	0.0658	0.0673	0.0425	0.1225
**IFNγ**	0.0972	1.0000	0.1993	0.0540	0.0880	-0.0717	-0.2116	0.1319	0.4395	-0.2009	0.2268
**IL-6**	0.0990	0.1993	1.0000	0.4051	0.3057	0.1540	0.3282	0.3264	0.3076	0.3206	0.2038
**GM-CSF**	0.0152	0.0540	0.4051	1.0000	0.1063	0.1197	0.0363	0.0991	0.3114	0.2240	0.0769
**IL-1β**	0.2593	0.0880	0.3057	0.1063	1.0000	0.2805	0.2312	0.2782	0.2529	0.2893	0.2741
**MCP-1**	0.2377	-0.0717	0.1540	0.1197	0.2805	1.0000	0.1693	0.4372	-0.0356	0.2543	0.4072
**MIP-1α**	0.0166	-0.2116	0.3282	0.0363	0.2312	0.1693	1.0000	0.6035	-0.0377	0.3864	0.3175
**MIP-1β**	0.0658	0.1319	0.3264	0.0991	0.2782	0.4372	0.6035	1.0000	0.1307	0.2774	0.6468
**IL-10**	0.0673	0.4395	0.3076	0.3114	0.2529	-0.0356	-0.0377	0.1307	1.0000	-0.0362	0.2042
**IP-10**	0.0425	-0.2009	0.3206	0.2240	0.2893	0.2543	0.3864	0.2774	-0.0362	1.0000	0.0944
**IL-17A**	0.1225	0.2268	0.2038	0.0769	0.2741	0.4072	0.3175	0.6468	0.2042	0.0944	1.0000

The table shows the data for Spearman's rank correlation coefficient (rho) or Spearman's *ρ*; A statistically significant level (p-value) for the Spearman's rank correlation coefficients is indicated in Table S6.

Our previous studies showed that overexpression of TILRR increased the secretion of several proinflammatory cytokine/chemokines.^2^ Thus, the positive correlation of plasma TILRR protein with the plasma proinflammatory cytokines/chemokines is consistent with TILRR promoting secretion of inflammatory cytokines/chemokines,^2^ and its potential influence on systemic inflammation.

### Plasma TILRR protein levels strongly predicted the risk of HIV seroconversion

**Figure 2:**
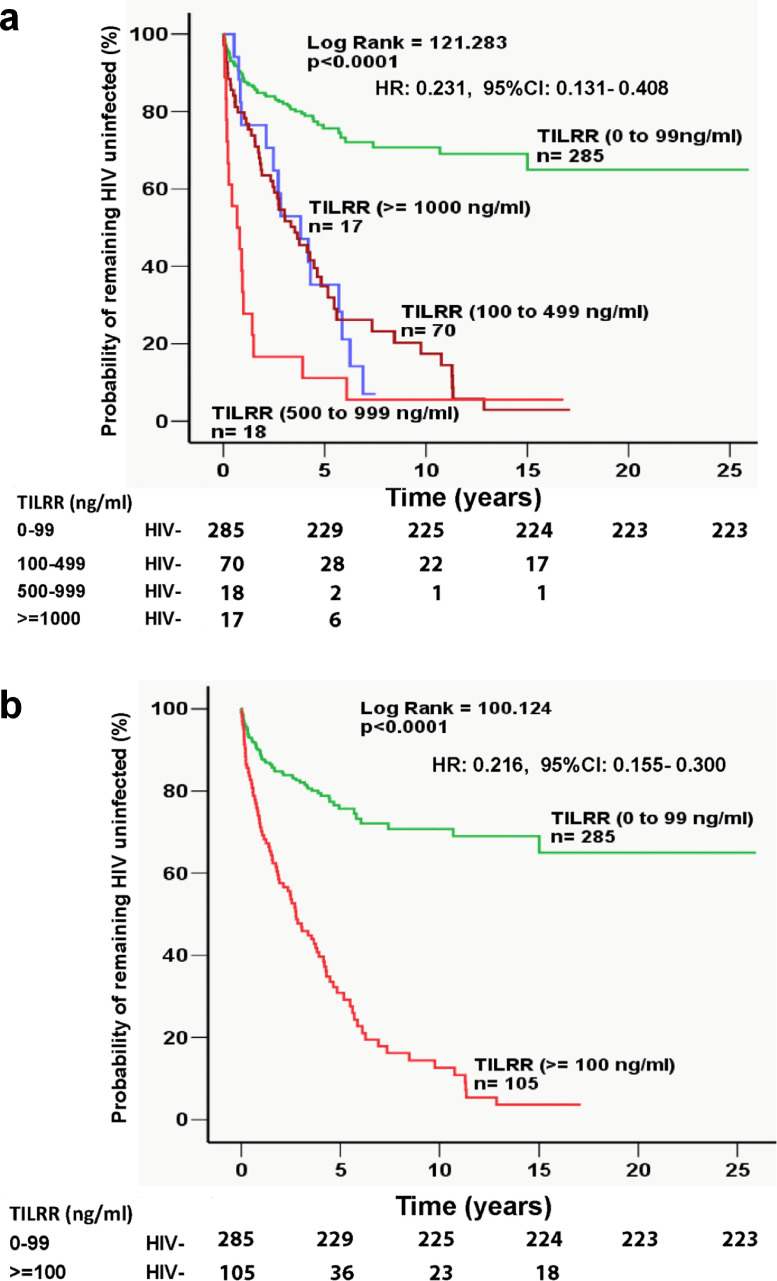
Kaplan-Meier survival analysis of plasma TILRR protein level and HIV seroconversion in the Pumwani sex worker cohort. (a) Sex workers who were HIV seronegative at enrolment of the Pumwani cohort were arbitrarily divided into 4 groups based on the median plasma TILRR protein level (0 to 99 ng/ml [green, *n*= 285], 100 to 499 ng/ml [dark red, *n*= 70], 500 to 999 ng/ml [red, *n*= 18] and those with plasma TILRR protein level ≥1000 ng/ml [blue, *n*= 17]). b) Sex workers were further divided into 2 groups based on their plasma TILRR protein level (<100 ng/ml [green, *n*= 285]) and those with plasma TILRR protein level ≥100 ng/ml [blue, n= 105]). Total HIV-negative participants are shown underneath the corresponding Kaplan-Meier survival curves. Cox regression hazard ratio (HR) with 95% CI is shown for the participants' group with 0-99 ng/ml plasma TILRR protein in both figures. Detailed point estimates for each Kaplan-Meier survival curve analysis are shown in Tables S7, 8. Statistical analysis was conducted using log-rank (Mantel-Cox). n, number of subjects; CI, confidence interval.

To determine the effect of plasma TILRR protein level on HIV seroconversion, the median value of the TILRR protein of multiple plasma samples collected at a different time from each woman was generated. The overall distribution of the median TILRR protein levels of the women was first analyzed, and then arbitrarily divided into four groups based on the TILRR protein levels in plasma (0-99 ng/ml, 100-499 ng/ml, 500-999 ng/ml, and ≥1000 ng/ml) (Figure 2a).

Kaplan-Meier survival analysis was conducted to evaluate the effect of median plasma TILRR protein levels on HIV seroconversion. The analysis showed that the three groups of women with median plasma TILRR protein levels at >99 ng/ml (100-499 ng/ml, 500-999 ng/ml, and ≥1000 ng/ml) were rapidly seroconverted after cohort enrolment (log rank [Mantel-Cox]: 121.283, *p*<0.0001) (Figure 2a and Tables S7, 8). Whereas women with the lowest median plasma TILRR protein level (0-99 ng/ml) were seroconverted significantly slower (Figure 2a). The group of women with the lowest median plasma TILRR protein level (0-99 ng/ml) remained seronegative for a prolonged period than the women with higher median TILRR protein (≥100 ng/ml) (log rank [Mantel-Cox]: 100.124, *p*<0.0001) (Figure 2b and Tables S7, 8).

Odds ratio analysis showed that women who had the median TILRR protein level in plasma ≥100 ng/ml were 15.29-fold more likely to become HIV infected, and their relative risk (RR) was 3.72 to the women who had median plasma TILRR protein level lower than 100 ng/ml (0-99 ng/ml) (Figure 2b). Thus, the TILRR protein levels in plasma strongly predicted the risk of HIV seroconversion. HIV-resistant women were more likely to have low median plasma TILRR levels and 95.9% of them (71/74) belong to the group of women with TILRR protein levels lower than 100 ng/ml.

### Age, duration of sex work and sexually transmitted infections (STIs) are similar among women with different levels of plasma TILRR protein

To examine whether there are statistically significant differences among the women with different levels of plasma TILRR protein (0-90 ng/ml, 100-499 ng/ml, 500-999 ng/ml, and ≥1000 ng/ml) in age, duration of sex work, STIs, vaginal discharge and genital ulcer, we analyzed the recorded clinical data at the sample collection (Table 1). STls, vaginal discharge, and genital ulcers of the subjects were included because these factors cause genital inflammation and increase the risk of HIV seroconversion.^4^^,^^9^ The analysis showed that there was no statistically significant difference between women with plasma TILRR protein level 0-99 ng/ml and women with plasma TILRR level ≥100 ng/ml (Tables 3 and S9). Thus, these factors were similar among women with different plasma TILRR protein levels.

**Table 3:** Comparison of potential confounding factors of study subjects with different plasma TILRR protein levels.

Characteristics	TILRR (0-99 ng/ml)Median (IQR)	TILRR (100-499 ng/ml)Median (IQR)	TILRR (500-999 ng/ml)Median (IQR)	TILRR (≥1000 ng/ml)Median (IQR)	Mean difference	p-value	95% CI
Age at sample collection (years)	34.00 (28.00-39.00) (n=273^a^)	31.00 (28.00-37.00) (n=65^a^)	32.00 (26.75-37.25) (n=16^a^)	33.00 (28.88-36.13) (n=16^a^)	2.1828^c^ 3.0150^d^ 2.0150^e^	0.1972^c^ 0.6797^d^ 1.0000^e^	-0.5205 to 4.8861^c^ -2.0233 to 8.0533^d^ -3.0233 to 7.0533^e^
Duration of sex work at sample collection (years)	8.00 (3.50-15.00) (n= 263^b^)	9.00 (4.00-12.00) (n= 65^b^)	7.00 (1.00-10.50) (n= 15^b^)	8.00 (4.25-12.50) (n= 15^b^)	1.2453^c^ 1.9043^d^ 0.4376^e^	1.0000^c^ 1.0000^d^ 1.0000^e^	-1.3992 to 3.8899^c^ -3.1639 to 6.9725^d^ -4.6306 to 5.5058^e^

	% (n)	% (n)	% (n)	% (n)	Pearson Chi-Square value		p-value
Sexually transmitted infection (STIs)	42.62 (119/279^f^)	52.17 (36/69^f^)	44.44 (8/18^f^)	47.06 (8/17^f^)	2.0303^g^ 0.0222^h^ 0.1270^i^		0.1542^g^ 0.8816^h^ 0.7215^i^
Vaginal discharge	28.67 (80/279^f^)	40.58 (28/69^f^)	27.78 (5/18^f^)	17.65 (3/17^f^)	3.6637^g^ 0.0066^h^ 0.9656i		0.0556^g^ 0.9350^h^ 0.3258^i^
Genital ulcer	10.75 (30/279^f^)	15.94 (11/69^f^)	16.67 (3/18^f^)	5.88 (1/17^f^)	1.4333^g^ 0.5988^h^ 0.4054^i^		0.2312^g^ 0.4390^h^ 0.5243 i

a Age at sample collection was unknown for 12-, 5-, 2 -, and 1-women with TILRR protein levels 0-99 ng/ml, 100-499 ng/ml, 500-999 ng/ml, and ≥1000 ng/ml, respectively.

b Duration of sex work at sample collection was unknown for 22-, 5-, 3-, and 2-women with TILRR protein levels 0-99 ng/ml, 100-499 ng/ml, 500-999 ng/ml, and ≥1000 ng/ml, respectively.

c One-way ANOVA followed by Bonferroni post-hoc test was conducted between women with TILRR protein levels 0-99 ng/ml and 100-499 ng/ml

d One-way ANOVA followed by Bonferroni post-hoc test was conducted between women with TILRR protein levels 0-99 ng/ml and 500-999 ng/ml

e One-way ANOVA followed by Bonferroni post-hoc test was conducted between women with TILRR protein levels 0-99 ng/ml and ≥1000 ng/ml

f Samples with a history of known STIs (Gonorrhea, Syphilis, Chlamydial infection, and bacterial vaginosis), vaginal discharge, and genital ulcer. Six women with TILRR protein level 0-99 ng/ml and one woman with TILRR protein level 100-499 ng/ml did not have a history of STIs, vaginal discharge, and genital ulcers.

g Chi-Square test conducted between women with TILRR protein levels 0-99 ng/ml and 100-499 ng/ml.

h Chi-Square test conducted between women with TILRR protein levels 0-99 ng/ml and 500-999 ng/ml.

i Chi-Square test conducted between women with TILRR protein levels 0-99 ng/ml and ≥1000 ng/ml IQR, interquartile range; n, subject#.

## Discussion

Our earlier studies showed that TILRR increases the expression of many genes in the NF-κB inflammation-signaling pathway, promotes the production of several proinflammatory mediators, and migration of immune cells.^2^^,^^3^^,^^19^ Recently, we showed that TILRR protein circulates in human blood.^20^ Because inflammation and proinflammatory mediators increase the risk of HIV acquisition,^4^^,^^7^^,^^9^^,^^27^^,^^28^ this study is to determine whether plasma TILRR levels correlate with plasma proinflammatory cytokines/chemokines and influence the susceptibility of HIV infection.

Pumwani sex worker cohort was established in 1985 in Nairobi, Kenya. Over >30 years of clinical follow-up, a subgroup of HIV-resistant female sex workers has been identified.^13^ The long-term clinical follow-up preserved clinical samples over years, including plasma samples collected since the cohort establishment. These archived plasma samples from women who were HIV negative at enrolment together with their clinical data were used for this study.

Consistent with our previous observation that TILRR promotes the secretion of many proinflammatory cytokines/chemokines,^2^ our study showed that the plasma TILRR protein level was positively correlated with the levels of plasma IL-1β, MCP-1, and IL-17A (Table 2). IL-1β is a pleiotropic cytokine, which mediates the expression of diverse cytokines/chemokines during the immune response and inflammation.^29^ Chemokine MCP-1 plays a critical role in recruiting CCR2 expressing memory CD4+T cells, and monocytes/macrophages.^30^ The recruitment of CD4+T cells and monocytes/macrophages by MCP-1 at the site of inflammation may fuel the HIV infection. Thus, it is possible that the high plasma TILRR protein level leads to increased plasma inflammatory cytokines/chemokines in these women. The high systemic inflammation may lead to higher immune activation status and increase the risk of HIV acquisition.^31^

This assumption is supported by the Kaplan-Meier seroconversion analysis (Figure 2a, b). Women with median plasma TILRR protein levels ≥100 ng/ml were rapidly seroconverted after cohort enrollment, and women with median plasma TILRR protein levels <100 ng/ml (0-99 ng/ml) remained HIV uninfected much longer (log rank [Mantel-Cox]: 100.124, *p*<0.0001; HR with 95% CI: 0.216 [0.155-0.300]) (Figure 2b). The factors causing genital inflammation and HIV seroconversion (age, STls, vaginal discharge, and genital ulcers)^4^^,^^9^^,^^32^ were not statistically significantly different among women with different median plasma TILRR protein levels (Tables 3 and S9). Thus, the high median plasma TILRR levels before seroconversion are strongly associated with the high risk of HIV seroconversion and can be used to predict the high risk of HIV acquisition.

In this study, the median levels of plasma TILRR protein were arbitrarily used to group the women based on the data distribution. The analysis turned out that median plasma TILRR protein level <100 ng/ml is a good indicator for low risk of HIV vaginal acquisition. Although TILRR is an important modulator of inflammatory responsive genes and promotes secretion of inflammatory cytokines/chemokines,^2^ how the 100 ng/ml plasma TILRR protein threshold defines the increased risk requires further investigation.

We previously showed that the mRNA expression of TILRR is absent or very low in women with the minor allele of FREM1 SNP rs1552896,^19^ but frequently observed in women who are homozygous for the major allele of SNP rs1552896.^10^ It is possible that the SNP rs1552896 may influence the splicing event of FREM1 and the generation of TILRR mRNA and TILRR protein in these women. This assumption is consistent with what we have observed for plasma TILRR protein level. Women with the minor allele of SNP rs1552896 were more likely to have low median plasma TILRR protein levels and 82.35% of them (14/17) have median plasma TILRR protein levels <100 ng/ml (Table S10). Because not all participants of this study have FREM1 SNP rs1552896 genotype data, we were unable to conduct a meaningful analysis for the association of plasma TILRR protein levels with FREM1 SNP rs1552896 genotype. These need to be further studied.

TILRR mRNA is highly expressed in immune cells and epithelial cell lines.^1^^,^^19^ Although TILRR promotes the production of many inflammatory mediators,^2^ the factors inducing the TILRR protein secretion from cells or tissues are not yet known. A study showed that the PBMCs (peripheral blood mononuclear cells) of patients diagnosed with atherosclerotic plaque and myocardial infarction expressed a higher level of TILRR protein.^33^ Thus, the secretion of TILRR protein may be induced by the injury or infection of the cells, and by the microbial products, such as lipopolysaccharide (LPS) and nucleic acids. Future studies are needed to identify the causal factors.

HIV-uninfected young men who have sex with men (MSM) showed an increased level of systemic inflammation^34^ and inflammation increases the risk of HIV infection.^4^^,^^9^ Because the plasma TILRR protein^20^ may increase many proinflammatory cytokines in blood,^1^ the higher systemic proinflammatory cytokines may also increase the risk of HIV acquisition in MSM. The current study is only possible because of the well-established and -followed Pumwani sex worker cohort that provided valuable clinical data since the beginning of the HIV epidemic and the archived biorepository of samples. Similar study could be conducted with other cohorts, including MSM cohort. The effect of plasma TILRR could also be investigated for HIV-associated co-morbidities that are associated with chronic inflammation and immune activation in People living with HIV (PLWH) despite under cART (combined antiretroviral therapy).^35^, ^36^, ^37^, ^38^ It would be interesting to study whether the level of plasma TILRR protein plays a role in HIV infection-associated chronic inflammation.

This study shows that the high plasma TILRR protein level statistically significantly correlated with increased susceptibility to HIV seroconversion. Because the cervicovaginal route is the major route of HIV transmission in the Pumwani sex worker cohort,^39^^,^^40^ how the plasma TILRR protein level directly influences the cervical-vaginal environment needs to be studied. The limitation of the study is the detection limit of the method we used, which was 2.38 ng/ml. It is possible that increasing the sensitivity of the detection of plasma TILRR protein can provide data for those who had very low plasma TILRR levels.

We did not introduce any known biases in any steps of the study, including data generation, curation, analysis, and presentation.

In summary, our study showed that plasma TILRR protein levels correlate with plasma proinflammatory cytokines/chemokines, and high plasma TILRR protein levels predicted the risk of HIV seroconversion, can possibly be used to predict HIV seroconversion risk in many populations. The study also showed that plasma TILRR protein may be a practical target to reduce systemic inflammation and the risk of HIV acquisition.

## Contributors

Mohammad Abul Kashem: conceived, designed, and performed the research, analyzed the data, wrote the manuscript, revised, formatted, and submitted to the journal; Jennifer Lischynski: performed research; Brittany Stojak: performed research; Lin Li: performed research and edited the manuscript; Xin-Yong Yuan: provided reagents; Binhua Liang: analyzed the data, reviewed and edited the manuscript; Joshua Kimani: maintained Pumwani sex worker cohort, coordinated the plasma sample collection and shipment, and ethics approval; Francis Plummer: established Pumwani sex worker cohort and maintained the cohort, acquired funding; Ma Luo: conceived and designed the research, analyzed the data, supervised the research, reviewed and edited the manuscript. Mohammad Abul Kashem and Ma Luo had full access to all the data in the study and have verified all the data. All authors have read and approved the final version of the manuscript.

## Declaration of interests

All authors declare that they have no conflict of interest. Dr. Plummer has nothing to disclose, filed on behalf author by Dr. Luo.

## Appendix Supplementary materials

Image, application 1
