# Supplementary material for: High level of plasma TILRR protein is associated with faster HIV seroconversion
Source: eBioMedicine. 2022 Mar 24;78:103955. doi: 10.1016/j.ebiom.2022.103955 (PMC8960884; doi:10.1016/j.ebiom.2022.103955)
Supplement: Supplementary file 1 [file mmc1.pdf]

Title: High level of plasma TILRR protein is associated with faster HIV seroconversion

Mohammad Abul Kashem[a,b,c,d], Jennifer Lischynski[e], Brittany Stojak[e], Lin Li[b,d], Xin-Yong Yuan[d], Binhua Liang[b,d,f], Joshua Kimani[a,g,h], Francis A Plummer[a†], Ma Luo[a,b,d*]

Authors' affiliation(s):
[a]Department of Medical Microbiology and Infectious Diseases, University of Manitoba, Winnipeg, MB, Canada.
[b]JC Wilt Infectious Diseases Research Centre, National Microbiology Laboratory, Public Health Agency of Canada Winnipeg, MB, Canada.
[c]Department of Microbiology and Veterinary Public Health, Chittagong Veterinary and Animal Sciences University, Chittagong, Bangladesh.
[d]National Microbiology Laboratory, Public Health Agency of Canada, Winnipeg, MB, Canada.
[e]The Max Rady College of Medicine, University of Manitoba, Winnipeg, MB, Canada.
[f]Department of Biochemistry & Medical Genetics, University of Manitoba, Winnipeg, MB, Canada.
[g]Institute for Tropical and Infectious Diseases, University of Nairobi, Nairobi, Kenya.
[h]Center for STD/HIV Research & Training, University of Nairobi

Running Title: Plasma TILRR protein and HIV seroconversion

†In Memoriam
*Correspondence to: Ma Luo, PhD
JC Wilt Infectious Diseases Research Center, National Microbiology Laboratory, 745 Logan Avenue, Winnipeg, MB, R3E 3L5, Canada;
Department of Medical Microbiology, University of Manitoba, Winnipeg, MB, Canada
Phone: 204-789-5072, Fax: 204-789-2018
Email: ma.luo@phac-aspc.gc.ca, Ma.Luo@umanitoba.ca

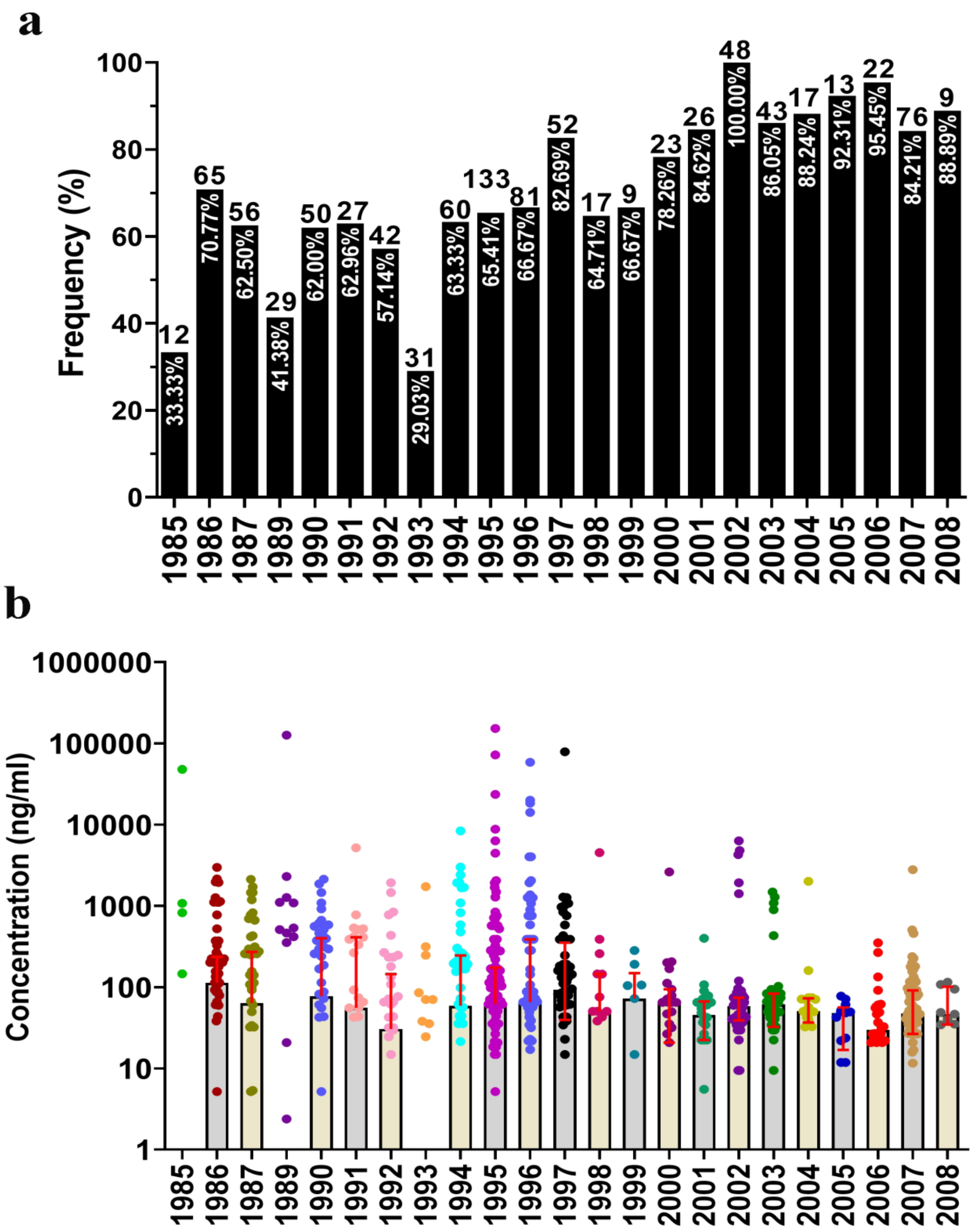

Figure S1: Frequency and the level of TILRR protein in archived plasma samples of all patients collected between 1985 and 2008. a) The frequency of plasma TILRR protein detection in archived plasma samples in different years between 1985 and 2008 (n=941). Value on the top of each bar represents the number of the sample (n) tested; **b)** The level of TILRR protein (median with IQR) in archived plasma samples collected between 1985 and 2008 (n=941). Detailed data are presented in Table S4. The X-axis indicates the collection year of the plasma samples.

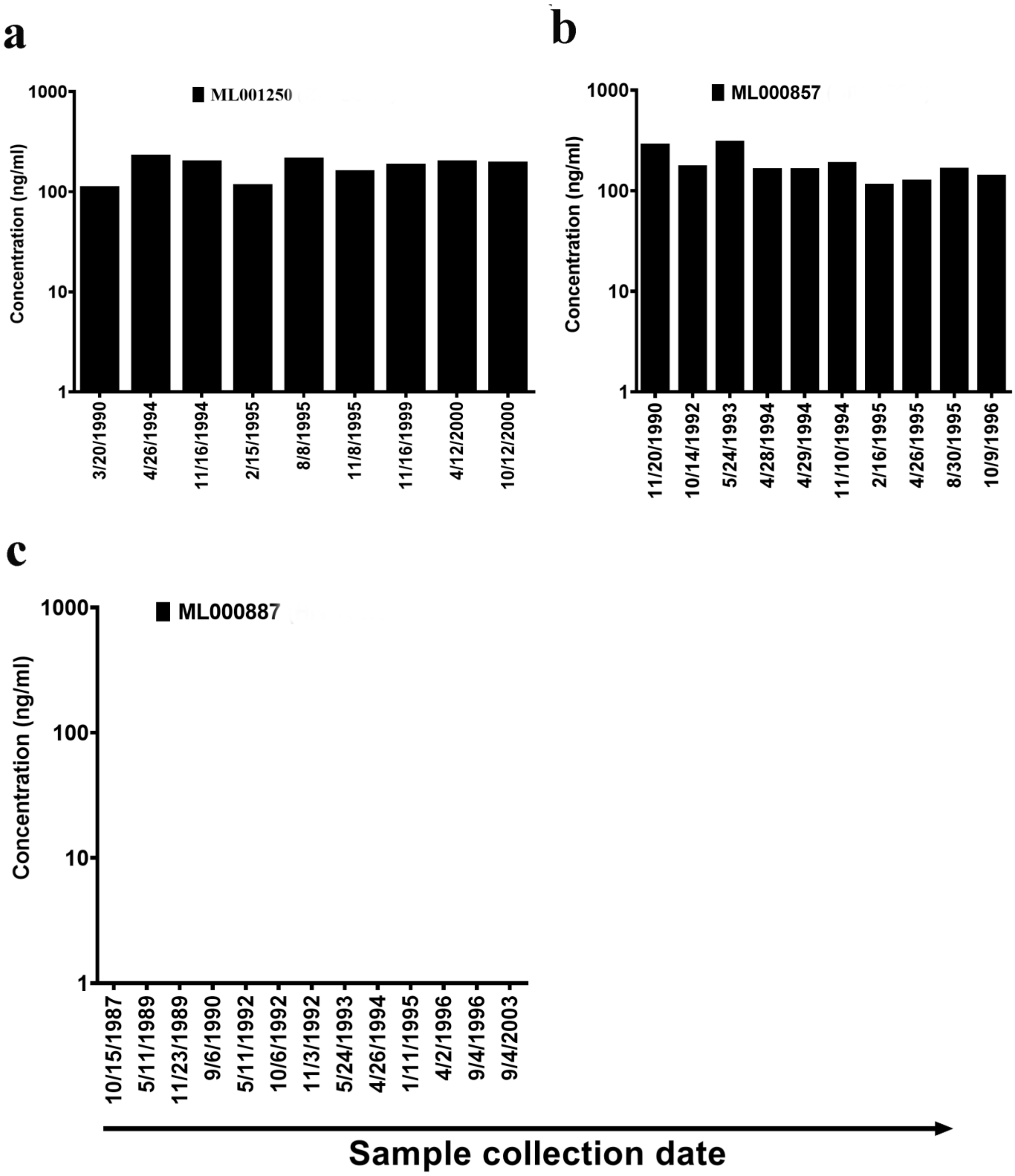

Figure S2: Level of plasma TILRR in archived samples collected from the same patient with multiple visits between 1987 and 2003. a-c) The level of plasma TILRR protein was quantified in three different women (ML001250, ML000857, and ML000887) of PSWC. In the case of figure **c**, TILRR levels were found below the limit of detection for participant ML000887. Detailed data are presented in Table S5. The X-axis indicates the sample collection date. Legend on the top of each figure shows the patient ID#.

Table S1: Power calculation for the study sample size using a post-hoc power calculator

Plasma TILRR level	Participants				Type I error rate (α)					Power of study (%)*
	HIV negative (n)	HIV seroconverted (n)	Total (n)	Infection risk (%)						
**Group 1 (0-99 ng/ml)**	223	62	285	21.75	0.05	0.01	0.001	0.0001	0.00001	100
**Group 2 (≥100 ng/ml)**	20	85	105	80.95						
**Overall**	**243**	**147**	**390**	**37.69**						
*For different type I error rates (α-value) with 390 sample size, a 100 % statistical study power was observed.										

Table S2: Primary and detection antibodies, and protein standards used in Bio-Plex Multiplex cytokines/chemokines bead assay

**Primary antibody (Uncoupled)**				
**SL**	**Name**		**Catalog#**	**Vendor**
**1**	Human IL-1 alpha/IL-1F1 Mab		MAB200	R&D system
**2**	Human IL-1beta/ IL-1F2 Antibody		MAB601-500	R&D System
**3**	Human IL-6 MAb		M620	ThermoFisher Scientific
**4**	Rat Anti-Human IL-10-UNLB		10100-01	SouthernBiotech
**5**	Human/Primate IL-17/IL-17A Antibody		MAB317-500	R&D System
**6**	Human IP-10/ CXCL10/CRG-2 Antibody		MAB266-500	R&D System
**7**	Human CXCL8/IL-8 MAb		M801	ThermoFisher Scientific
**8**	Human CCL5/RANTES PAb		P230E	ThermoFisher Scientific
**9**	Rat Anti-Human GM-CSF-UNLB		10111-01	SouthernBiotech
**10**	Human IFNγ MAb		M700A	ThermoFisher Scientific
**11**	Human MCP-1/CCL2/JE Antibody		MAB679-500	R&D System
**12**	Human MIP-1α/CCL3Antibody		AF-270-NA	R&D System
**13**	Human MIP-1β/CCL4 Antibody		MAB271-100	R&D System
**14**	Human TNFα MAb		M303	ThermoFisher Scientific
**Detection antibody (Biotinylated)**				
**SL#**	**Name**		**Catalog#**	**Vendor**
**1**	Human IL-1 alpha/IL-1F1 Biotinylated		BAF200	R&D System
**2**	Human IL-1beta/IL-1F2 Biotinylated Antibody		BAF201	R&D System
**3**	Human IL-6 MAb, Biotin-labeled		M621B	ThermoFisher Scientific
**4**	Rat Anti-Human IL-10-BIOT		10110-08	SouthernBiotech
**5**	Human/Primate IL-17/IL-17A Biotinylated Antibody		BAF317	R&D System
**6**	Human IP-10/CXCL10/CRG-2 Biotinylated Antibody		BAF266	R&D System
**7**	Human CXCL8/IL-8 MAb, Biotin-labeled		M802B	ThermoFisher Scientific
**8**	Human CCL5/RANTES MAb, Biotin-labeled		M230B	ThermoFisher Scientific
**9**	Rat Anti-Human GM-CSF-BIOT		10112-08	SouthernBiotech
**10**	Human IFNγ MAb, Biotin-labeled		M701B	ThermoFisher Scientific
**11**	Human MCP-1/CCL2/JE Biotinylated Antibody		BAF279	R&D System
**12**	Human MIP-1α/CCL3 Biotinylated Antibody		BAF270	R&D System
**13**	Human MIP-1β/CCL4 Biotinylated Antibody		BAF271	R&D System
**14**	Human TNFα MAb, Biotin-labeled		M302B	ThermoFisher Scientific
**Cytokine/chemokine protein standards**				
**SL#**	**Name**	**Final pool concentration (pg/ml)**	**Catalog#**	**Vendor**
**1**	Recombinant Human IL-1 alpha	160000	200-LA	R&D System

**2**	Recombinant Human IL-1 beta/IL-1F2	19500	201-LB	R&D System
**3**	Recombinant Human IL-6 Protein	9500	206-IL-10	R&D System
**4**	Recombinant Human IL-10 (aa 19-178) Protein	28000	1064-IL	R&D System
**5**	Recombinant Human IL-17A Protein	38000	317-ILB	R&D System
**6**	Recombinant Human IP-10/CXCL10	24000	266-IP	R&D System
**7**	Recombinant Human CXCL8/IL-8 Protein	27000	208-IL-10	R&D System
**8**	Recombinant Human CCL5/RANTES Protein	18500	278-RN-10	R&D System
**9**	Recombinant Human GM-CSF Protein	17000	215-GM	R&D System
**10**	Recombinant Human Interferon Gamma Protein	10500	RIFNG50	ThemoFisher Scientific
**11**	Recombinant Human CCL2/MCP-1 Protein	26500	279-MC	R&D System
**12**	Recombinant Human CCL3/MIP-1 alpha protein	27500	270-LD-10	R&D System
**13**	Recombinant Human CCL4/MIP-1 beta Protein	9000	271-BME-10	R&D System
**14**	Recombinant Human TNF-alpha Protein	18000	210-TA-20	R&D System
**A part of this table is adapted from Kashem et al.[1] under the license CC BY 4.0**				

Table S3: Frequency and level of TILRR protein in the available archived plasma samples of all HIV uninfected women at enrolment of PSWC

**Frequency of plasma TILRR protein detection**						
**Year**	**1985-1990**	**1991-1995**	**1996-2000**	**2001-2005**	**2006-2008**	**Total plasma samples (n)**
**TILRR+ (n)**	128	175	132	134	93	**662**
**TILRR- (n)**	84	118	50	13	14	**279**
**Total plasma samples (n)**	**212**	**293**	**182.00**	**147**	**107**	**941**
**Frequency (%)**	**60.38**	**59.73**	**72.53**	**91.16**	**86.92**	

**Level of plasma TILRR protein**					
	**2006-2008**	**2001-2005**	**1996-2000**	**1991-1995**	**1985-1990**
**Mean**	95.40	224.71	1315.86	1155.73	1113.51
**SE**	26.72	64.87	561.56	582.82	635.47
**Median**	46.45	51.57	66.11	43.36	75.65
**Stdev**	276.36	786.54	7575.80	9976.24	9252.63
**Minimum**	0.00	0.00	0.00	0.00	0.00
**Maximum**	2795.83	6326.60	79041.61	152872.63	126307.51
**Sum**	10207.58	33031.90	239486.00	338627.66	236063.79
**95% CI:**					
**Lower bound**	42.43	96.50	207.82	8.67	-139.18
**Upper bound**	148.37	352.92	2423.89	2302.78	2366.20

Table S4: Frequency and the level of TILRR protein in archived plasma samples of all patients collected between 1985 and 2008

Frequency of plasma TILRR protein detection

**Year**	**1985**	**1986**	**1987**	**1989**	**1990**	**1991**	**1992**	**1993**	**1994**	**1995**	**1996**	**1997**
**TILRR+**(n)	4	46	35	12	31	17	24	9	38	87	54	43
**TILRR-**(n)	8	19	21	17	19	10	18	22	22	46	27	9
**Total** (n)	**12**	**65**	**56**	**29**	**50**	**27**	**42**	**31**	**60**	**133**	**81**	**52**
**%Frequency**	**33.33**	**70.77**	**62.50**	**41.38**	**62.00**	**62.96**	**57.14**	**29.03**	**63.33**	**65.41**	**66.67**	**82.69**

**Year**	**1998**	**1999**	**2000**	**2001**	**2002**	**2003**	**2004**	**2005**	**2006**	**2007**	**2008**	**Total (n)**
**TILRR+** (n)	11	6	18	22	48	37	15	12	21	64	8	**662**
**TILRR-** (n)	6	3	5	4	0	6	2	1	1	12	1	**279**
**Total** (n)	**17**	**9**	**23**	**26**	**48**	**43**	**17**	**13**	**22**	**76**	**9**	**941**
**%Frequency**	**64.71**	**66.67**	**78.26**	**84.62**	**100.00**	**86.05**	**88.24**	**92.31**	**95.45**	**84.21**	**88.89**	

Level of plasma TILRR protein

**Year**	**1985**	**1986**	**1987**	**1989**	**1990**	**1991**	**1992**	**1993**	**1994**	**1995**	**1996**	**1997**
**Mean**	4166.73	334.86	271.12	4633.51	294.86	362.45	176.12	84.49	464.72	2187.54	1696.98	1759.12
**SE**	3982.03	73.61	63.14	4346.64	66.69	190.91	60.82	56.42	157.96	1278.03	803.68	1516.09
**Median**	0.00	113.38	63.75	0.00	77.17	55.61	30.65	0.00	59.02	61.52	65.44	92.95
**Stdev**	13794.15	593.49	472.48	23407.36	471.60	992.01	394.14	314.15	1223.57	14738.90	7233.12	10932.70
**Minimum**	0.00	0.00	0.00	0.00	0.00	0.00	0.00	0.00	0.00	0.00	0.00	0.00
**Maximum**	47953.57	2975.23	2120.21	126307.50	2131.30	5196.79	1928.23	1732.33	8373.19	152872.60	58564.11	79041.61
**Sum**	50000.79	21765.82	15182.50	134371.80	14742.89	9786.14	7397.07	2619.14	27883.03	290942.30	137455.00	91474.27
**95% CI:**												
**Lower bound**	-4597.66	187.80	144.59	-4270.17	160.83	-29.98	53.30	-30.74	148.63	-340.52	97.60	-1284.56
**Upper bound**	12931.12	481.92	397.65	13537.19	428.89	754.88	298.94	199.72	780.80	4715.60	3296.35	4802.80

**Year**	**1998**	**1999**	**2000**	**2001**	**2002**	**2003**	**2004**	**2005**	**2006**	**2007**	**2008**	
**Mean**	338.97	85.69	174.91	57.44	438.98	164.31	169.49	40.04	64.65	108.72	58.07	
**SE**	262.45	32.84	111.91	14.87	185.95	52.50	115.05	6.70	18.13	37.19	12.95	
**Median**	50.36	72.44	65.03	45.22	58.36	61.37	50.36	48.13	29.78	47.69	46.48	
**Stdev**	1082.11	98.51	536.68	75.82	1288.31	344.28	474.38	24.16	85.05	324.24	38.85	
**Minimum**	0.00	0.00	0.00	0.00	9.45	0.00	0.00	0.00	0.00	0.00	0.00	
**Maximum**	4518.06	282.00	2621.42	399.54	6326.60	1484.55	2005.21	77.61	351.37	2795.83	115.28	
**Sum**	5762.50	771.18	4022.96	1493.53	21071.13	7065.30	2881.40	520.54	1422.19	8262.77	522.62	
**95% CI:**												
**Lower bound**	-217.40	9.96	-57.17	26.82	64.89	58.35	-74.41	25.44	26.94	34.63	28.21	
**Upper bound**	895.34	161.41	406.99	88.07	813.07	270.26	413.40	54.64	102.35	182.81	87.93	

This table is split up for both %frequency and concentration of TILRR protein due to the long period (1985 to 2008)

Table S5: Level of plasma TILRR in archived samples collected from the same patient with multiple visits between 1987 and 2003

ML001250		ML000857		ML000887*	
**Sample collection date (mm/dd/yyyy)**	**TILRR (ng/ml)**	**Sample collection date (mm/dd/yyyy)**	**TILRR (ng/ml)**	**Sample collection date (mm/dd/yyyy)**	**TILRR (ng/ml)**
**3/20/1990**	113.73	**11/20/1990**	294.65	**10/15/1987**	0
**4/26/1994**	233.72	**10/14/1992**	179.32	**5/11/1989**	0
**11/16/1994**	205.40	**5/24/1993**	314.96	**11/23/1989**	0
**2/15/1995**	119.04	**4/28/1994**	167.09	**9/6/1990**	0
**8/8/1995**	247.73	**4/29/1994**	167.09	**5/11/1992**	0
**11/8/1995**	164.32	**11/10/1994**	192.08	**10/6/1992**	0
**11/16/1999**	191.02	**2/16/1995**	117.44	**11/3/1992**	0
**4/12/2000**	205.21	**4/26/1995**	128.36	**5/24/1993**	0
**10/12/2000**	200.42	**8/30/1995**	168.61	**4/26/1994**	0
		**10/9/1996**	143.74	**1/11/1995**	0
				**4/2/1996**	0
				**9/4/1996**	0
				**9/4/2003**	0

*In the case of ML000887, TILRR levels were found below the limit of detection

Table S6: Spearman rank correlation of plasma TILRR with plasma inflammatory cytokines/chemokines (n=352)

	TILRR	IFNγ	IL-6	GM-CSF	IL-1β	MCP-1	MIP-1α	MIP-1β	IL-10	IP-10	IL17A
**TILRR (rho)**	1.0000	0.0972	0.0990	0.0152	0.2593	0.2377	0.0166	0.0658	0.0673	0.0425	0.1225
**p-value**	.	0.0685	0.0635	0.7765	**<0.0001**	**<0.0001**	0.7566	0.2179	0.2076	0.4266	**0.0216**
**IFNγ (rho)**	0.0972	1.0000	0.1993	0.0540	0.0880	-0.0717	-0.2116	0.1319	0.4395	-0.2009	0.2268
**p-value**	0.0685	.	**0.0002**	0.3121	0.0994	0.1793	**<0.0001**	**0.0133**	**<0.0001**	**0.0001**	**<0.0001**
**IL-6 (rho)**	0.0990	0.1993	1.0000	0.4051	0.3057	0.1540	0.3282	0.3264	0.3076	0.3206	0.2038
**p-value**	0.0635	**0.0002**	.	**<0.0001**	**<0.0001**	**0.0038**	**<0.0001**	**<0.0001**	**<0.0001**	**<0.0001**	**0.0001**
**GM-CSF(rho)**	0.0152	0.0540	0.4051	1.0000	0.1063	0.1197	0.0363	0.0991	0.3114	0.2240	0.0769
**p-value**	0.7765	0.3121	**<0.0001**	.	**0.0464**	**0.0247**	0.4978	0.0633	**<0.0001**	**<0.0001**	0.1500
**IL-1β (rho)**	0.2593	0.0880	0.3057	0.1063	1.0000	0.2805	0.2312	0.2782	0.2529	0.2893	0.2741
**p-value**	**<0.0001**	0.0994	**<0.0001**	**0.0464**	.	**<0.0001**	**<0.0001**	**<0.0001**	**<0.0001**	**<0.0001**	**<0.0001**
**MCP-1 (rho)**	0.2377	-0.0717	0.1540	0.1197	0.2805	1.0000	0.1693	0.4372	-0.0356	0.2543	0.4072
**p-value**	**<0.0001**	0.1793	**0.0038**	**0.0247**	**<0.0001**	.	**0.0014**	**<0.0001**	0.5051	**<0.0001**	**<0.0001**
**MIP-1α (rho)**	0.0166	-0.2116	0.3282	0.0363	0.2312	0.1693	1.0000	0.6035	-0.0377	0.3864	0.3175
**p-value**	0.7566	**<0.0001**	**<0.0001**	0.4978	**<0.0001**	**0.0014**	.	**<0.0001**	0.4804	**<0.0001**	**<0.0001**
**MIP-1β (rho)**	0.0658	0.1319	0.3264	0.0991	0.2782	0.4372	0.6035	1.0000	0.1307	0.2774	0.6468
**p-value**	0.2179	**0.0133**	**<0.0001**	0.0633	**<0.0001**	**<0.0001**	**<0.0001**	.	**0.0142**	**<0.0001**	**<0.0001**
**IL-10 (rho)**	0.0673	0.4395	0.3076	0.3114	0.2529	-0.0356	-0.0377	0.1307	1.0000	-0.0362	0.2042
**p-value**	0.2076	**<0.0001**	**<0.0001**	**<0.0001**	**<0.0001**	0.5051	0.4804	**0.0142**	.	0.4986	**0.0001**
**IP-10 (rho)**	0.0425	-0.2009	0.3206	0.2240	0.2893	0.2543	0.3864	0.2774	-0.0362	1.0000	0.0944
**p-value**	0.4266	**0.0001**	**0.0001**	**<0.0001**	**<0.0001**	**<0.0001**	**<0.0001**	**<0.0001**	0.4986	.	0.0769
**IL-17A (rho)**	0.1225	0.2268	0.2038	0.0769	0.2741	0.4072	0.3175	0.6468	0.2042	0.0944	1.0000
**p-value**	**0.0216**	**<0.0001**	**0.0001**	0.1500	**<0.0001**	**<0.0001**	**<0.0001**	**<0.0001**	**0.0001**	0.0769	.

rho, Spearman's rank correlation coefficient or Spearman's ρ; Statistically significant p-values showed as bold
This table is included as a supplementary table for the information of p-values.

Table S7: Means and Medians of HIV negative time for the 4 groups of women defined by the plasma TILRR protein level (1985-2008)

Means and Medians related to figure 2A						
Plasma TILRR level (ng/ml)	Mean HIV negative time			Median HIV negative time		
	Mean	Std. Error	95% CI	Median	Std. Error	95% CI
**0-99**	18.377	0.925	16.563 to 20.191	.	.	.
**100-499**	4.715	0.590	3.558 to 5.872	3.526	0.682	2.189 to 4.863
**500-999**	1.979	0.917	0.182 to 3.777	0.677	0.386	0.000 to 1.434
**≥1000**	3.775	0.557	2.684 to 4.867	3.825	1.017	1.832 to 5.818

Means and Medians related to figure 2B						
Plasma TILRR level (ng/ml)	Mean HIV negative time			Median HIV negative time		
	Mean	Std. Error	95% CI	Median	Std. Error	95% CI
**0-99**	18.377	0.925	16.563 to 20.191	.	.	.
**≥100**	4.115	0.457	3.220 to 5.011	2.748	0.463	1.841 to 3.655
**Overall**	**13.222**	**0.813**	**11.628 to 14.815**	**9.767**	**1.735**	**6.366 to 13.168**

Table S8: HIV infection risk of the 4 groups defined by the plasma TILRR protein level

HIV infection risk related to figure 2A				
Plasma TILRR level (ng/ml)	Participants			
	HIV negative (n)	HIV seroconverted (n)	Total (n)	Infection risk (%)
**0-99**	223	62	285	21.75
**100-499**	17	53	70	75.71
**500-999**	1	17	18	94.44
**≥1000**	2	15	17	88.24
**Overall**	**243**	**147**	**390**	**37.69**

Overall Comparisons			
	Chi-Square	df	p-value
**Log Rank (Mantel-Cox)**	121.2827	3	4.08483E-26

HIV infection risk related to figure 2B				
Plasma TILRR level (ng/ml)	Participants			
	HIV negative (n)	HIV seroconverted (n)	Total (n)	Infection risk (%)
**0-99**	223	62	285	21.75
**≥100**	20	85	105	80.95
**Overall**	**243**	**147**	**390**	**37.69**

Overall Comparisons			
	Chi-Square	df	p-value
**Log Rank (Mantel-Cox)**	100.1240	1	1.4315E-23

Table S9: Comparison of confounding factors of study subjects with different plasma TILRR protein levels

Characteristics	TILRR (0-99 ng/ml) Median (IQR)	TILRR (≥100 ng/ml) Median (IQR)	Mean difference	p-value	95% CI
**Age at sample collection (years)**	34.00 (28.00-39.00) (n=273[a])	32.00 (28.00-37.00) (n=97[a])	2.2924[c]	0.0088[c]	0.5802[c] to 4.0045
**Duration of sex work at sample collection (years)**	8.00 (3.50-15.00) (n= 263[b])	8.00 (4.00-12.00) (n= 95[b])	-0.0750[c]	0.9332[c]	-1.8336[c] to 1.6836
	**% (n)**	**% (n)**	**Pearson Chi-Square value**		**p-value**
**Sexually transmitted infection (STIs)**	42.62 (119/279[d])	50.00 (52/104[d])	1.6550[e]		0.1983[e]
**Vaginal discharge**	28.67 (80/279[d])	34.62 (36/104[d])	1.2667[e]		0.2604[e]
**Genital ulcer**	10.75 (30/279[d])	13.46 (14/104[d])	0.9843[e]		0.3211[e]

[a]Age at sample collection was unknown for 12- and 8-women with TILRR protein levels 0-99 ng/ml and ≥100 ng/ml, respectively.
[b]Duration of sex work at sample collection was unknown for 22- and 10-women with TILRR protein levels 0-99 ng/ml and ≥100 ng/ml, respectively.
[c]t-test was conducted between women with TILRR protein levels 0-99 ng/ml and ≥100 ng/ml
[d]Samples with a history of known STIs (Gonorrhea, Syphilis, Chlamydial infection, and bacterial vaginosis), vaginal discharge, and genital ulcer. Six women with TILRR protein level 0-99 ng/ml and one woman with TILRR protein level 100-499 ng/ml did not have a history of STIs, vaginal discharge, and genital ulcers.
[e]Chi-Square test conducted between women with TILRR protein levels 0-99 ng/ml and ≥100 ng/ml
IQR, interquartile range; n, subject#

Table S10: Relationship of plasma TILRR protein with FREM1 SNP rs1552896 genotypes

FREM1 rs1552896 genotypes	Participants (n)	Plasma TILRR protein level	
		**Mean ± Stdev**	**Median (IQR)**
**Minor allele (GG/GC genotype)**	17	49.11±49.54	40.86 (1.19-67.97)
**Major allele (CC genotype)**	169	104.73±246.99	46.45 (17.12-80.95)
**Total**	186	-	-

REFERENCES

1. Kashem MA, Li H, Toledo NP, et al. Toll-like Interleukin 1 Receptor Regulator Is an Important Modulator of Inflammation Responsive Genes. Frontiers in Immunology. 2019;10(272):1-16. DOI: 10.3389/fimmu.2019.00272.
